# Exercise Training in Patients with Established Cancer Cachexia: A Case Series

**DOI:** 10.3390/healthcare14213601

**Published:** 2026-10-23

**Authors:** Christian Ciurnelli, Andrea Trevisan, Anita Borsati, Arianna Giorgetti, Gloria Adamoli, Ilaria Trestini, Daniela Tregnago, Jordan Curry, Thomas D. Cardaci, Michele Milella, Federico Schena, Sara Pilotto, Alice Avancini

**Affiliations:** 1 Department of Neurosciences, Biomedicine and Movement, University of Verona, P. le L.A. Scuro 10, 37129 Verona, Italy; 2 Biomedical, Clinical and Experimental Sciences, Department of Medicine, University of Verona, 37129 Verona, Italy; 3 Section of Innovation Biomedicine—Oncology Area, Department of Engineering for Innovation Medicine (DIMI), University of Verona and University and Hospital Trust (AOUI) of Verona, 37129 Verona, Italy; 4 Department of Inflammation, Immunity and Cancer, University of Verona, 37129 Verona, Italy; 5 Dietetic Service, Medical Direction, Azienda Ospedaliera Universitaria Integrata di Verona, 37129 Verona, Italy; 6 Department of Health and Exercise Science, Colorado State University, Fort Collins, CO 80523, USA; 7 Hull York Medical School, University of Hull, Hull HU6 7RX, UK; 8 Human Performance Research Centre, INSIGHT Research Institute, Faculty of Health, University of Technology Sydney (UTS), Sydney 2007, NSW, Australia; 9 Department of Medicine, Division of Medical Oncology, University of Colorado Anschutz Medical Campus, Aurora, CO 80045, USA; 10 Comprehensive Cancer Center, University of Colorado, Aurora, CO 80045, USA

**Keywords:** cachexia, cancer, structured exercise, physical fitness, body composition, quality of life

## Abstract

**Background/Objectives:** Exercise is a supportive strategy for patients with cancer; however, evidence in individuals with established cancer cachexia remains limited. This study aimed to provide a preliminary description of feasibility and exploratory changes during a supervised exercise program in patients with cancer cachexia. **Methods**: Five women diagnosed with gastrointestinal or breast cancer and meeting the diagnostic criteria for cancer cachexia participated in a 12-week supervised exercise program, combining aerobic with resistance training, conducted twice weekly. Feasibility outcomes included completion, attendance, adherence, and adverse events. Muscle strength, flexibility, body composition, functional capacity, and patient-reported outcomes were assessed at baseline and post-intervention. **Results:** All participants completed the intervention and post-intervention assessments. Attendance ranged from 54% to 96%, with interruptions primarily related to treatment schedules and treatment-related side effects. No serious exercise-related adverse events occurred. Functional capacity improved in all participants (+ 33 to +73 m). Grip strength increased in all patients for the right hand (+0.5 to +5.5 kg) and in three patients for the left hand (+2.0 to +3.5 kg). Body composition responses were heterogeneous: fat-free mass increased in three patients (+1.3 to +2.8 kg), whereas two patients showed reductions. Fat mass decreased in three patients (−2.6 to −5.0 kg) and increased in two. Several domains of quality of life and symptom burden improved in most participants. **Conclusions:** In these five women with cancer cachexia, a supervised exercise program showed variable attendance and no serious exercise-related adverse events, whereas favorable changes for functional outcomes and heterogeneous response in body composition were observed.

## 1. Introduction

An international consensus has defined cancer cachexia as “*a multifactorial syndrome characterized by an ongoing loss of skeletal muscle mass (with or without loss of fat mass) that cannot be fully reversed by conventional nutritional support and leads to progressive functional impairment*” [1]. Cancer cachexia affects approximately 33% of the oncological population; however, its prevalence varies according to cancer type and stage, with advanced-stage and upper-gastrointestinal cancers showing the highest incidence [2]. Cachexia remains a major clinical challenge since it is associated with impaired treatment tolerance and efficacy, as well as poor survival. In this regard, a meta-analysis, including 137,960 patients with cancer, estimated that cachectic patients had a 58% higher mortality risk compared with their non-cachectic counterparts [2].

Therefore, interventions aimed at preventing, managing, and potentially reversing cachexia are fundamental. To date, no pharmacological therapies have been approved in Europe or the United States for the treatment of cancer-related cachexia [3], while Anamorelin has been approved in Japan [4]. However, recently promising results have been observed for ponsegromab [5]. However, given the complexity of cancer cachexia, international guidelines strongly recommend the integration of supportive care interventions as part of its management [6]. Beyond nutritional support, which is considered crucial in this context, the introduction of exercise has also been advocated [6].

Exercise may act on several consequences of cancer cachexia, including skeletal muscle wasting, bone demineralization, metabolic alterations, cardiac dysfunction, microbiome dysregulation, and alteration of the anorexigenic and orexigenic pathways. Furthermore, exercise may modulate key mechanisms involved in cachexia initiation and progression, such as the chronic systemic inflammatory milieu driven by pro-inflammatory cytokines and cachexia-inducing factors (CIFs), tumor-derived extracellular vesicles and miRNAs that propagate inflammatory and metabolic dysregulation and contribute to muscle damage, and the activity of tumor microenvironment (TME) cells (e.g., macrophages, neutrophils, fibroblasts, and other immune cells) that sustain cytokine and chemokine release, chronic inflammation and impaired muscle regeneration [7].

By improving physical fitness and attenuating fatigue, exercise may counteract the progressive decline in functional capacity. Additionally, exercise may positively influence psychological well-being and social engagement, potentially mitigating distress, anxiety, and depressive symptoms, social isolation, and the impaired quality of life (QoL) typically observed in patients with cachexia [8].

Despite this strong biological and clinical rationale, evidence supporting exercise interventions specifically in patients with cancer cachexia remains limited. A Cochrane systematic review identified only four small studies and concluded that the available evidence is insufficient to determine the benefits or risks of exercise, alone or in combination with other interventions, in adults with cancer cachexia [9]. Furthermore, two recent systematic reviews have summarized the effects of exercise in patients considered at high risk of developing cachexia (e.g., those with lung, gastrointestinal, or head and neck cancers). However, in most cases, the included studies involved populations with a normal body mass index (BMI), without documented weight loss and without a formal diagnosis of cancer cachexia [10,11].

Consequently, studies specifically analyzing the effect of exercise in patients with established cachexia remain scarce. Therefore, the present case series aims to describe the feasibility and exploratory individual changes in clinical and functional outcomes of a structured exercise intervention in patients with cancer affected by cachexia.

## 2. Materials and Methods

### 2.1. Study Design and Participants

The present study reports a consecutive case series of five women with established cancer cachexia who were referred to the exercise program during the study period and met the eligibility criteria. The study aimed to describe the feasibility and exploratory clinical and functional outcomes of a structured exercise program. No otherwise eligible patients were excluded because of incomplete training or follow-up, and the inclusion of women only was not an eligibility criterion but reflects the consecutive cases identified during the study period.

Participants were diagnosed with gastrointestinal cancer (*n* = 3 stage IV; *n* = 1 stage III) and breast cancer (*n* = 1 stage III). According to the Fearon criteria [1], all participants exhibited unintentional weight loss of ≥ 2% in the previous 6 months with a BMI < 20 kg/m^2^, or unintentional weight loss of ≥ 5% in the previous 6 months, thus indicating cachexia. Historical weight loss was assessed by the dietitian from body weight measurements documented in the clinical records during the preceding six months as part of the baseline nutritional evaluation.

All patients were receiving nutritional care at the Oncology Section of the Hospital Trust and were referred by the dietitian between October 2021 and September 2024 to participate in the exercise program. Nutritional care included assessment and counseling by a registered dietitian, with the goals of providing patients and caregivers with practical and safe advice on feeding; education on high-protein, high-calorie, and nutrient-dense foods; and guidance against fad diets and other unproven or extreme dietary approaches. This intervention was consistent with the recommendations of the American Society of Clinical Oncology (ASCO) guideline on the management of cancer cachexia [3].

Patients who expressed interest were contacted by the study staff, provided detailed information about the study, and asked to obtain medical clearance prior to participation. Written informed consent was obtained from each participant before any study procedures.

This case series was conducted within the larger project approved by the Ethics Committee for Clinical Trials of the University of Verona (protocol no. 33320, dated 17 June 2019) and prospectively registered on ClinicalTrials.gov (NCT04226508). The project includes different patient cohorts investigated using different study designs; this specific cachexia case series was not separately prespecified in the trial registration, and the cases included in the present report were not previously published. This case series was reported in accordance with the CARE guidelines (Supplementary Table S3) [12].

### 2.2. Exercise Intervention

The exercise intervention was based on the current available guidelines for patients with cancer from the American College of Sports Medicine (ACSM) [13] and Exercise & Sports Science Australia (ESSA) [14]. Because these recommendations are intended for the general cancer population, and patients with cachexia are typically more deconditioned and present distinctive clinical and metabolic features, additional considerations were incorporated into the exercise prescription based on the available literature on this topic [11].

Among the key considerations, a combined aerobic and resistance training approach was selected. Aerobic exercise was included to promote systemic anti-inflammatory effects and preserve cardiorespiratory fitness while carefully avoiding excessive energy expenditure (e.g., limiting exercise duration). Resistance training was incorporated to stimulate muscle protein synthesis and counteract muscle wasting, given its anabolic and anti-catabolic effects [15]. Moreover, both exercise modalities may influence the tumor microenvironment through immune and metabolic adaptations [7].

Given the severe deconditioning and metabolic vulnerability typical of cancer cachexia, a cautious and individualized progression, together with strict monitoring of exercise intensity, was adopted to minimize the risk of disproportionate muscle damage associated with excessive exercise [15].

Based on these premises, the exercise intervention was conducted at the facilities of the Sport Science Section of the University of Verona. The program, delivered twice weekly for 12 weeks, was supervised by dedicated exercise professionals with prior experience working with patients with cancer and was individualized according to each patient’s clinical history and physical and psychological condition. Before each exercise session, participants underwent a verbal symptom check to assess their current clinical condition, including perceived fatigue and any new or worsening symptoms.

Each session consisted of combined aerobic and resistance training. After 5 min of warm-up (dynamic stretching), aerobic training on a cycloergometer was performed. The individualized starting duration ranged from 10 to 15 min/session according to baseline functional capacity and clinical condition. Aerobic duration was progressively increased according to a predefined schedule, provided that perceived exertion remained within the prescribed range, up to a maximum achieved duration of 20 min/session. Exercise intensity was prescribed at RPE 3–5 on the modified Borg CR-10 scale, a category-ratio scale anchored at 10 to represent maximal exertion [16]. Resistance training consisted of five exercises per session selected from a predefined pool targeting the major muscle groups. Exercise selection varied across sessions according to functional capacity, symptoms, clinical condition, and available range of motion. The individualized starting volume ranged from 2 × 8 to 2 × 10 repetitions, and was progressively increased according to a predefined schedule every two weeks up to a maximum achieved volume of 5 exercises performed for 3 × 12 repetitions. Progression was implemented only when perceived exertion remained within the prescribed range. Rest periods of 1–2 min between sets were provided. External resistance was individually adjusted according to perceived exertion, set at 3–5 on the Borg Scale [16], and clinical tolerance; absolute external loads were not systematically recorded. Each session concluded with a 5 min cool-down comprising static stretching exercises. Planned increases in aerobic duration and resistance-training volume represented predefined progression of the exercise prescription and were distinguished from unplanned dose modifications required because of symptoms or exercise intolerance. Case-specific starting doses, progression, maximum achieved doses, exercise selection, and individual adaptations are reported in Supplementary Table S1.

The initial aerobic exercise intensity was adjusted according to the participant’s reported fatigue and perceived exertion, generally starting at a light intensity. Throughout the session, aerobic and resistance exercise intensity was monitored using the RPE scale and participants’ reported symptoms. When perceived exertion exceeded the prescribed range (RPE 6–7), exercise intensity was reduced by adjusting the cyclergometer’s intensity during aerobic training or by decreasing the external load during resistance training. Exercise was temporarily interrupted when necessary to allow symptom reassessment before determining whether the session could be resumed at a lower intensity or should be postponed. For patient #1, who had a history of cardiomyopathy, and patient #4, who had hypertension, exercise intensity was individualized with particular attention to perceived exertion, fatigue, and cardiovascular symptoms (e.g., palpitations).

### 2.3. Outcomes

Feasibility outcomes included completion rate, attendance, adherence and safety. Study completion was defined as the number of participants who completed both baseline and post-intervention assessments, irrespective of the number of exercise sessions attended. The intervention consisted of 24 planned sessions, delivered twice weekly over 12 consecutive weeks. Participants who were unable to attend a scheduled session were offered the opportunity to reschedule it within the same week; otherwise, sessions were recorded as missed and were not carried over to subsequent weeks. The attendance rate was calculated as the number of sessions attended divided by the 24 planned sessions, expressed as a percentage. Attendance was categorized as dropout (permanent discontinuation), missed sessions (1–2 consecutive missed sessions), or interruption (≥3 consecutive missed sessions) [17]. Adherence to the prescribed exercise dose during attended sessions was defined as the proportion of attended sessions in which the prescribed exercise session was completed as planned. Adherence was also characterized by dose modifications, defined as any session requiring an increase or decrease in the prescribed exercise dosage, or early session termination before reaching the target intensity or duration [18]. Safety was monitored throughout the study period. Exercise-related adverse events were defined as new or worsening symptoms occurring during supervised exercise sessions and considered attributable to exercise. Events were recorded by the supervising exercise professional when they occurred and graded according to the Common Terminology Criteria for Adverse Events (CTCAE), version 5.0 [19]. Treatment-related symptoms that prevented attendance at scheduled sessions were recorded separately as reasons for missed visits.

A series of standardized physical fitness assessments and patient-reported outcomes were administered at baseline and post-intervention. Baseline and post-intervention assessments were performed by the same assessor using the same procedures, with post-intervention testing conducted within one week of the last exercise session. Assessments were conducted at least 48 h after the last exercise session to minimize the influence of acute exercise-related changes. Functional capacity was assessed using the six-minute walking test (6MWT) conducted according to the American Thoracic Society guidelines [20], without prior familiarization testing. Upper-body muscle strength was evaluated using maximal voluntary isometric contraction with a Hydraulic Hand Dynamometer (Model SH5001, Saehan Corporation, South Korea), and the highest value from five attempts was recorded [21].

Upper- and lower-limb flexibility were assessed using the back scratch test and the chair sit-and-reach test, respectively; two attempts were performed for each test [22]. Anthropometric parameters, including body weight, height, body mass index, and hip and waist circumferences, were measured according to the World Health Organization procedures [23]. Whole-body fat-free mass and fat mass were assessed using bioimpedance analysis (Tanita RD-545 HR) [24]. Participants were assessed barefoot and wearing light clothing, after emptying their bladder, and were instructed to maintain their usual hydration status and to avoid exercise before testing. Measurements were performed under similar environmental conditions, including room temperature, at baseline and post-intervention. Information on the presence of edema or ascites and on the timing of bioimpedance measurements relative to systemic treatment was not systematically collected.

Patient-reported outcomes included quality of life, assessed using the Italian version 3.0 of the European Organization for Research and Treatment of Cancer Quality of Life Questionnaire Core (EORTC-QLQ-C30) [25]. The EORTC QLQ C-30 comprises 30 items organized into five functional scales (physical, role, cognitive, emotional, and social), three symptom scales (fatigue, pain, and nausea and vomiting), and a global health and quality-of-life scale. Additional single items were used to assess symptoms commonly reported by cancer patients, such as dyspnea, sleep disturbance, appetite loss, constipation, and diarrhea. The scores range from 0 to 100, with higher scores indicating better functioning and global health status, whereas higher scores on symptom scales indicate greater symptom burden. No questionnaire items were missing, and scores were calculated according to the EORTC QLQ-C30 Scoring Manual [26].

### 2.4. Statistical Analysis

Data are presented case by case for baseline characteristics and outcome measures at each time point. Changes over time were described by calculating absolute and relative percentage differences between baseline and post-intervention for the objective outcomes.

To enhance interpretability, changes in quality-of-life scores were expressed as absolute score point differences between post-intervention and baseline values (T1–T0).

Descriptive statistics were used to assess the feasibility and safety of the intervention.

## 3. Results

The baseline characteristics of the participants are presented in Table 1. Briefly, the sample included five women aged 28 to 60 years, with a diagnosis of pancreatic (*n* = 3), stomach (*n* = 1), and breast (*n* = 1) cancer. The patients had a mean body mass index of 19.1 kg/m^2^ and a mean unintentional weight loss of 15% over the previous 6 months. All patients were undergoing systemic treatments.

### 3.1. Attendance, Adherence, and Adverse Events

**Figure 1:**
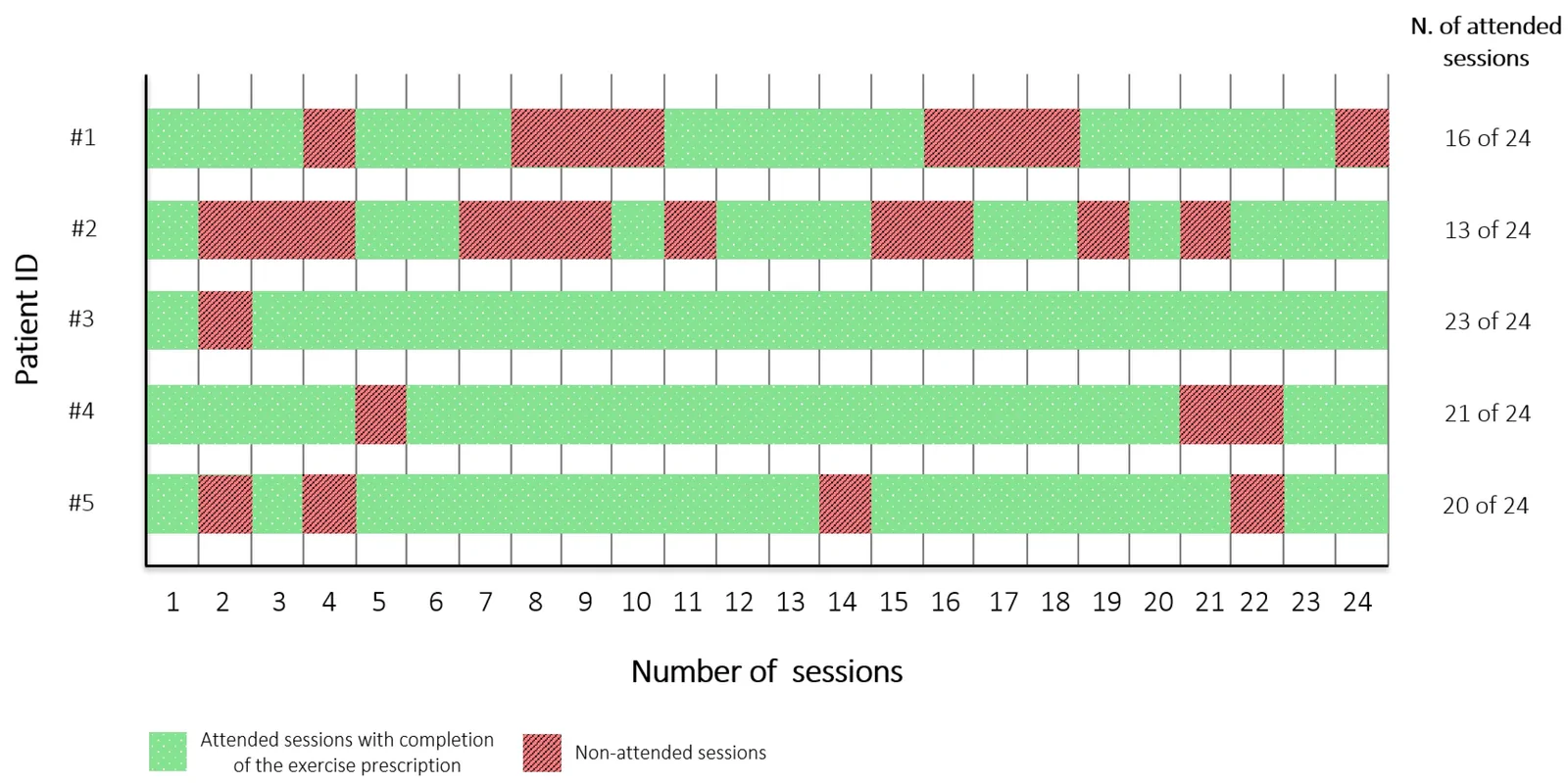
Individual attendance across the 24 prescribed exercise sessions during the 12-week intervention. Each column represents one scheduled session. Dotted blocks indicate attended sessions with completion of the prescribed exercise session, while diagonally hatched blocks indicate non-attended sessions. Numbers on the right indicate the total sessions attended by each participant (n = 5) out of 24 prescribed sessions.

All patients completed baseline and post-intervention evaluations, and no dropouts occurred. Figure 1 illustrates attendance and adherence to the exercise prescription.

Attendance exceeded 80% for patients #3 (23/24 scheduled sessions; 95.8%), #4 (21/24 scheduled sessions; 87.5%), and #5 (20/24 scheduled sessions; 83.3%). Attendance below 70% was observed for patients #1 (16/24 scheduled sessions; 66.7%) and #2 (13/24 scheduled sessions; 54.2%). Exercise interruptions occurred twice in both participants #1 and #2. Participant #1 missed three consecutive sessions due to surgery (sessions 8–10) and three consecutive sessions due to treatment-related side effects (sessions 16–18). Participant #2 experienced two interruptions, both attributable to treatment-related side effects (sessions 2–4 and 7–9). Only one session was rescheduled during the study period. No participant required extension of the intervention beyond the planned 12 calendar weeks.

Overall, 27 sessions were missed due to concomitant oncological visits, surgery or treatment administration (*n* = 13), treatment-related side effects (*n* = 9), and personal reasons (*n* = 5). All attended sessions were completed as prescribed, with no unplanned dose modifications or early session terminations. Consequently, adherence to the prescribed exercise dose during attended sessions was 100% for all participants.

Six grade 1 adverse events occurred during supervised exercise sessions over the 12-week intervention period: five episodes of muscle soreness (patient #2, *n* = 2; patient #3, *n* = 1; patient #4, *n* = 2) and one episode of dizziness (patient #3). All events were resolved within the same day or the following day and did not require medical intervention or modification of the exercise program.

### 3.2. Physical Fitness and Patients’ Reported Outcomes

**Figure 2:**
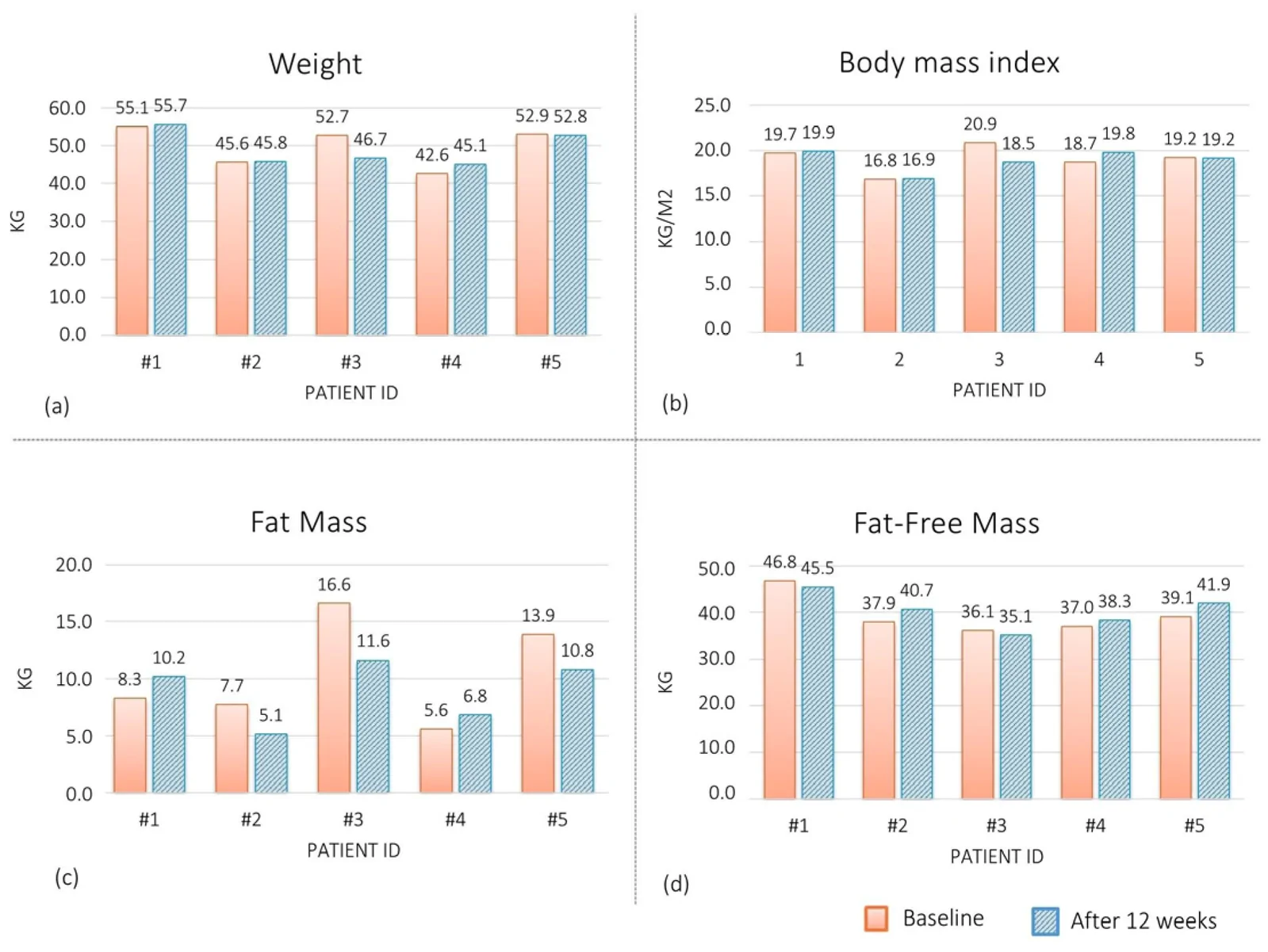
Individual changes in body composition from baseline to the end of the 12-week exercise intervention. (**a**) Body weight (kg); (**b**) body mass index (kg/m^2^); (**c**) fat mass (kg); and (**d**) fat-free mass (kg). Solid bars represent baseline values, whereas diagonally hatched bars represent values assessed after 12 weeks. Abbreviations and units: kg, kilograms; kg/m^2^, kilograms per square meter.

Anthropometric and body composition parameters are presented in Figure 2 and in Supplementary Table S2. At post-intervention, body weight and BMI remained stable in patients #1, #2, and #5. An increase was observed in patient #4 (+2.5 kg; +1.1 kg/m^2^), whereas a marked decrease was detected in patient #3 (−6 kg; −2.2 kg/m^2^). In patient #3, this additional weight loss corresponded to approximately 11.4% of baseline body weight, was accompanied by a reported decrease in energy intake, and prompted nutritional reassessment.

Fat mass decreased in patients #2 (−2.6 kg), #3 (−5.0 kg), and #5 (−3.1 kg), and increased in patients #1 (+1.9 kg) and #4 (+1.2 kg). A gain in fat-free mass was observed in patients #2 (+2.8 kg), #4 (+1.3 kg), and #5 (+2.8 kg), whereas reductions were detected in patients #1 (−1.3 kg) and #3 (−1.0 kg). The waist–hip ratio decreased in patients #2, #3, and #4, increased in patient #5, and remained stable in patient #1 (Figure S1).

**Figure 3:**
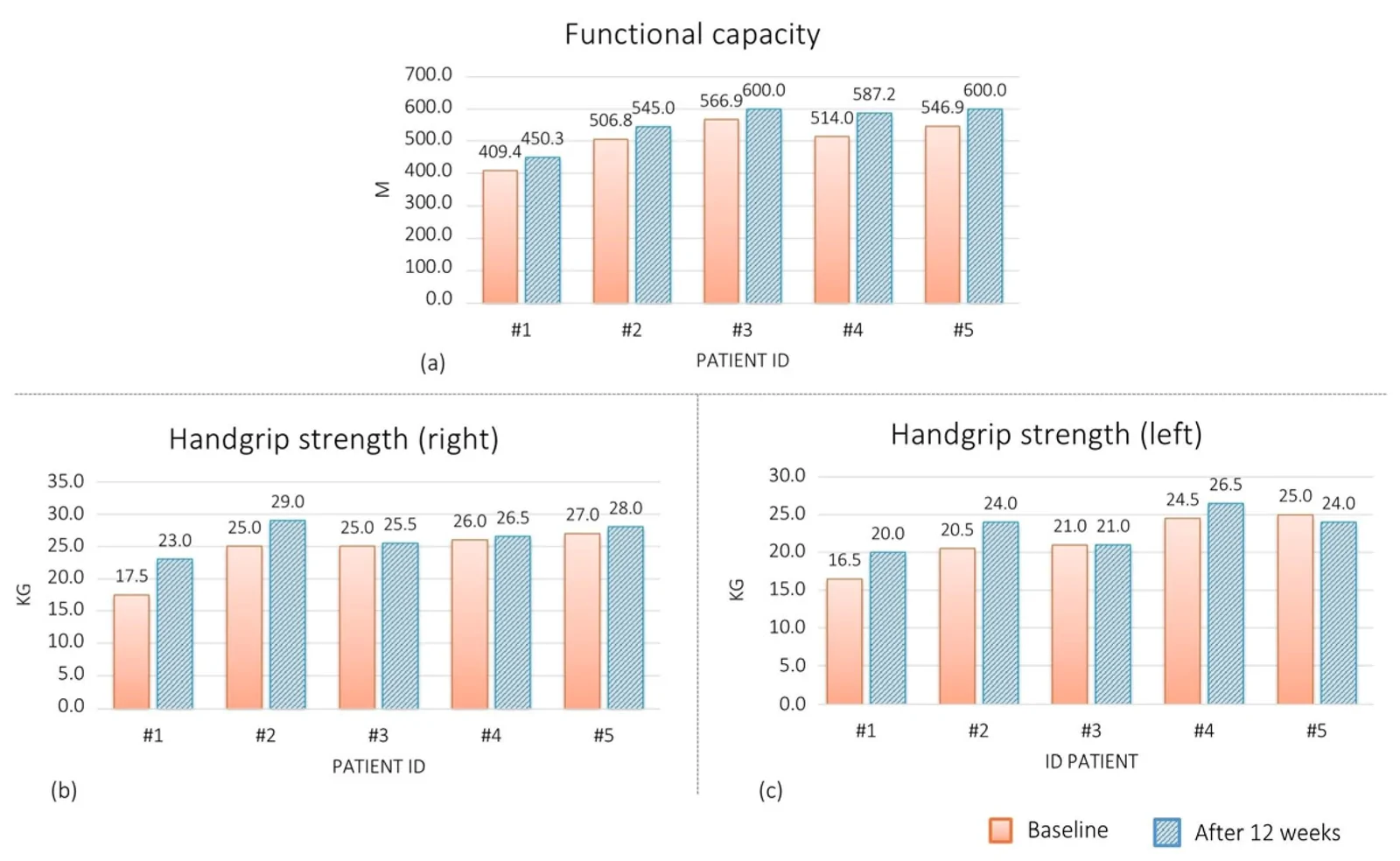
Individual changes in functional capacity and muscle strength from baseline to the end of the 12-week exercise intervention. (**a**) Functional capacity (m); (**b**) right-hand handgrip strength (kg); and (**c**) left-hand handgrip strength (kg). Solid bars represent baseline values, whereas diagonally hatched bars represent values assessed after 12 weeks. Abbreviations and units: kg, kilograms; m, meters.

Functional capacity improved in all patients, ranging from 33 to 73 m (Figure 3 and Supplementary Table S2). Grip strength increased in all patients for the right hand (ranging from +0.5 kg to 5.5 kg). For the left hand, improvements were observed in patients #1 (+3.5 kg), #2 (+3.5 kg), and #4 (+2.0 kg), whereas a decrease was observed in patient #5 (−1.0 kg), and values remained stable in patient #3 (Figure 3 and Supplementary Table S2). Flexibility measures are reported in Figure S2. Lower-limb flexibility improved in both legs in participants #1, #2, #4, #5, while it decreased in both legs in participant #3. Upper-limb flexibility of the right arm improved in patients #1, #2, #4, and #5, whereas improvements in the left arm were observed in patients #2 and #4.

Table 2 reports baseline and post-intervention measures of patient-reported outcomes. Global health status remained stable or improved in four patients (patients #1, #3, #4, and #5) and deteriorated by 8.3 points in patient #2.

Across the functioning scales, improvements were observed in most domains across participants, except for role functioning (patient #3), emotional functioning (patients #2 and #4), and social functioning (patients #3 and #4), which showed deterioration.

Among the symptom scales, fatigue improved in patients #1, #2, #4, and #5. Nausea and vomiting improved in patient #1 but worsened in patient #3. Pain and dyspnea worsened in patient #3. Insomnia improved in patients #2 and #4 and worsened in patient #1. Diarrhea improved in patient #5 and deteriorated in patients #2 and #4. Financial difficulties worsened in patients #3 and #4 and improved in patient #5.

## 4. Discussion

In this case series of five women with established cancer cachexia defined according to the Fearon diagnostic criteria [1], a 12-week supervised combined aerobic–resistance exercise program delivered twice weekly was successfully implemented. In this light, no participants withdrew, all completed baseline and post-intervention assessments, and adverse events were mild (grade 1 muscle soreness and transient dizziness) without the need for program modification. Although no serious exercise-related adverse events were observed among these five participants, the small sample size precludes broader conclusions regarding the safety of exercise in patients with cancer cachexia. These findings align with previous evidence indicating that tailored exercise interventions are generally safe in individuals with cachexia, with low rates of exercise-related adverse events and clinically acceptable levels of completion and adherence when programs are individualized [10,11,27].

Although all five participants completed the study, attendance varied considerably, with two patients attending fewer than 70% of the scheduled sessions. Missed sessions were primarily attributable to oncological visits, treatment administration, and treatment-related side effects rather than exercise-related intolerance. This variability in attendance represents an important feasibility finding, suggesting that, although completion of a structured exercise program may be possible, maintaining consistent participation can be challenging in the context of ongoing cancer treatment and symptom burden. This pattern mirrors the broader advanced and palliative cancer exercise literature, in which attendance rates of 60–75% are commonly observed, while attrition and interruptions often reflect disease burden and treatment demands rather than harms related to the intervention [28,29,30,31]. Importantly, the present program incorporated symptom-responsive progression and perceived exertion monitoring, which are consistent with recommendations from cachexia reviews emphasizing cautious exercise dosing, flexibility around “bad days”, and integration with supportive care to maintain feasibility [10,30]. These findings further highlight the potential value of flexible scheduling and, where appropriate, home-based or hybrid delivery models to accommodate treatment burden and logistical constraints.

Across all five cases, 6MWT distance increased from baseline to post-intervention (individual changes ranging from +33 to +73 m), alongside generally favorable changes in grip strength and flexibility. However, these observed pre–post changes cannot be attributed specifically to the exercise intervention, given the absence of a control group and the potential influence of concurrent oncological treatments, nutritional care, and individual clinical trajectories. In cachexia, where a progressive decline in strength and physical performance is expected, maintenance or improvement in functional outcomes may be clinically meaningful even in the absence of weight gain [32,33]. Preservation of functional capacity is particularly relevant in this population, as declines in muscle strength and physical function are strongly associated with treatment intolerance, loss of independence, and reduced survival in advanced cancer. Recent cachexia-focused evidence suggests that the most consistent functional benefits occur when programs include a resistance component, with clearer effects on muscle strength than on QoL outcomes, which tend to be more heterogeneous [11]. The individual functional trajectories observed in the present case series are encouraging, but should be interpreted as preliminary, hypothesis-generating findings rather than evidence of intervention efficacy.

Body weight and body composition responses were heterogeneous: three participants maintained body weight and BMI, one showed increases in both measures, three demonstrated gains in fat-free mass assessed by bioimpedance, and one experienced marked weight loss. Notably, the participant with the greatest weight loss had undergone gastrectomy, which promotes weight reduction through reduced gastric capacity and early satiety, accelerated gastrointestinal transit, impaired nutrient digestion and absorption, and postoperative complications such as nausea, vomiting, dumping syndrome, diarrhea, and steatorrhea [34].

These mechanisms could independently drive postoperative weight loss and amplify vulnerability to cachexia during ongoing anticancer therapy. In this context, functional preservation (or improvement) may represent a more realistic short-term therapeutic goal than weight gain, highlighting why weight stabilization, when achieved, may represent a clinically relevant observation rather than an expected outcome of the exercise intervention.

From a physiological perspective, the potential role of exercise in cancer cachexia is supported by several mechanisms described in the previous literature. Cancer cachexia is characterized by systemic inflammation, metabolic disruption, and dysregulated protein turnover that contribute to anabolic resistance, muscle wasting, and functional decline [33,35]. Aerobic and resistance exercises may counteract these processes through enhanced immune function, improved mitochondrial content and function, and stimulation of anabolic signaling pathways (e.g., Akt/mTOR) that regulate muscle protein synthesis. These proposed mechanisms provide a biological rationale for investigating whether exercise may contribute to the preservation of functional capacity and skeletal muscle mass in patients with established cachexia [33,35]. Although this case series did not assess inflammatory or metabolic biomarkers, the observed preservation or improvement in functional capacity provides preliminary observations that warrant further investigation in larger controlled studies.

The present observations may also inform future research investigating the integration of exercise within multimodal cachexia management strategies. Current guidelines emphasize multimodal care (including nutrition, symptom management, and selected pharmacologic strategies) and have historically highlighted the lack of sufficient evidence to formally recommend exercise in cachexia, identifying an important research gap [3,6]. Case studies [36] and preliminary data reported in a conference abstract from the MENAC trials [37] have investigated the implementation of home-centered mixed-modality exercise alongside nutritional support and anti-inflammatory medication, with preliminary evidence suggesting potential benefits for weight-related outcomes [37]. Although the present program was supervised and delivered in a facility-based setting, it reflects the same underlying principle: combined aerobic–resistance training integrated within supportive care, with flexible progression to accommodate treatment burden.

Finally, variability in attendance across cases is consistent with behavioral evidence indicating that symptom burden and psychosocial determinants strongly influence physical activity engagement in advanced cancer. In this series, EORTC QLQ-C30 scores reflected heterogeneous changes in symptom burden and social functioning, suggesting that symptoms such as fatigue, pain, dyspnea, and nausea may constrain both participation and adherence to exercise. Constructs such as affective attitude (immediate perceived benefit), self-efficacy, perceived behavioral control, autonomous motivation, and social support are consistently associated with physical activity engagement, whereas pain, fatigue, breathlessness, fear of overexertion, and uncertainty regarding exercise safety represent major barriers [38,39,40,41,42]. From a practical perspective, this supports the design of cachexia exercise programs that are autonomy-supportive, explicitly address safety concerns, incorporate collaborative goal setting and self-monitoring, and allow symptom-responsive adaptations. Home-centered or hybrid delivery models may further reduce geographical or logistical barriers to participation [40,43]. Future studies involving well-phenotyped individuals meeting the diagnostic criteria for cancer cachexia should prospectively assess these behavioral determinants alongside adherence, symptom trajectories, and patient-centered functional outcomes to better understand who benefits most and under what conditions.

Several limitations should also be acknowledged. First, the small sample size, absence of a control group, and uncontrolled case series design preclude causal inference regarding the effects of exercise and limit the generalizability of the findings. The inclusion of women only, together with the heterogeneity of cancer diagnoses, disease stages, treatment histories, and ongoing systemic therapies, further limits the applicability of these observations to the wider cancer cachexia population. Moreover, all participants were receiving nutritional care and systemic cancer treatment, which may have influenced their individual clinical trajectories. Second, potential selection bias should be considered, as participants were referred by a dietitian and subsequently expressed interest in participating in the exercise program. Furthermore, as this exploratory case series was not designed as a formal feasibility trial, recruitment-related data were not systematically collected. The absence of routine, structured screening for cancer cachexia in our Oncology Unit prevented the reliable identification of the total number of potentially eligible patients; consequently, the number of patients approached and the reasons for non-participation could not be reliably determined. Third, the substantial variability in attendance limits conclusions regarding the feasibility of delivering the full prescribed intervention. Similarly, although no serious exercise-related adverse events were observed, the inclusion of only five participants limits the ability to characterize intervention safety or identify uncommon adverse events. Lastly, blood- or tissue-based biomarkers were not assessed, limiting insight into the biological pathways through which exercise may influence systemic or skeletal muscle biology in cachectic patients. In addition, body composition was assessed using bioelectrical impedance analysis, which may be influenced by hydration status and may introduce measurement variability. Larger prospective studies incorporating systematic recruitment monitoring, molecular outcomes, and comprehensive feasibility assessments alongside behavioral and clinical measures are needed to better characterize the feasibility, safety, and potential effects of exercise in patients with cancer cachexia.

## 5. Conclusions

Collectively, this case series provides preliminary feasibility and safety observations for a 12-week supervised aerobic and resistance exercise program in women with established cancer cachexia, with no dropouts and only mild adverse events. Improvements in functional capacity were observed in all participants, while weight and body composition changes were variable, reflecting the heterogeneity of patient responses and the complex clinical context of cachexia. These observations support further evaluation of individualized, symptom-responsive exercise as a potential component of multimodal supportive care for patients with established cancer cachexia. Larger controlled studies are required to determine feasibility across broader patient populations, efficacy, safety, and optimal exercise prescription.

## Figures and Tables

**Table 1:** Demographic and clinical characteristics of the cases.

Variables	*Ca* *se #1*	*Case #2*	*Case #3*	*Case #4*	*Case #5*
Age (years)	59	52	28	55	60
BMI (kg/m^2^)	19.7	16.8	20.9	18.7	19.2
Unintentional weight loss in the previous 6 months (%)	24.0	19.8	9.3	7.0	14.8
Educational level	High school degree	Bachelor’s degree	High school degree	High school degree	Bachelor’s degree
Marital status	Married	Married	Married	Married	Married
Employment status	Retired	Retired	Retired	Homemaker	Retired
Financial resources	Barely adequate	Adequate	Adequate	Barely adequate	Very easily
Cancer site	Breast	Pancreas	Stomach	Pancreas	Pancreas
Months since diagnosis	27	13	41	12	3
Cancer stage (metastatic site)	III	IV (Lung)	IV (Peritoneum; Ovary)	IV (Liver)	III
Previous treatments (type)	Yes (chemoimmunotherapy, mastectomy, radiotherapy)	Yes (chemotherapy)	Yes (chemotherapy, gastrectomy)	Yes (chemotherapy)	Yes (chemotherapy)
Current treatment (type)	Yes (Immunotherapy—pembrolizumab)	Yes (Chemotherapy—folfirinox)	Yes (Chemotherapy—folfox)	Yes (Chemotherapy—folfirinox)	Yes (Chemotherapy—folfirinox)
Comorbidity (type)	Yes (Cardiomyopathy)	No	No	Yes (Hypertension)	No
Concurrent medication	Beta-blockers	None	None	Statin	None
Clinical course during follow-up	Performance status stable; systemic treatment unchanged; nutritional care continued	Performance status stable; systemic treatment unchanged; nutritional care continued	Performance status stable; systemic treatment unchanged; 6 kg weight loss associated with decreased energy intake; nutritional reassessment performed	Performance status stable; systemic treatment unchanged; nutritional care continued	Performance status stable; systemic treatment unchanged; nutritional care continued

**Table 2:** Changes in quality of life.

Va riables	*Case* *#1*			*Case #2*			*Case #3*			*Case #4*			*Case #5*		
	*T0*	*T1*	Δ *Score (T1–T0)*	*T0*	*T1*	Δ *Score (T1–T0)*	*T0*	*T1*	Δ *Score (T1–T0)*	*T0*	*T1*	Δ *Score (T1–T0)*	*T0*	*T1*	Δ *Score (T1–T0)*
EORTC QLQ-C30															
Global health status	16.7	41.7	25.0	58.3	50.0	−8.3	50.0	50.0	0.0	50.0	50.0	0.0	83.3	100.0	16.7
Physical functioning	46.7	66.7	20.0	86.7	93.3	6.7	86.7	93.3	6.7	86.7	93.3	6.7	93.3	100.0	6.7
Role functioning	33.3	50.0	16.7	66.7	100.0	33.3	100.0	66.7	−33.3	83.3	83.3	0.0	66.7	100.0	33.3
Emotional functioning	25.0	41.7	16.7	91.7	83.3	−8.3	66.7	75.0	8.3	91.7	75.0	−16.7	75.0	75.0	0.0
Cognitive functioning	33.3	50.0	16.7	100.0	100.0	0.0	83.3	100.0	16.7	66.7	83.3	16.7	66.7	100.0	33.3
Social functioning	0.0	50.0	50.0	66.7	100.0	33.3	83.3	66.7	−16.7	100.0	66.7	−33.3	16.7	83.3	66.7
Fatigue	55.6	44.4	−11.1	33.3	11.1	−22.2	33.3	33.3	0.0	33.3	22.2	−11.1	33.3	11.1	−22.2
Nausea and vomiting	66.7	16.7	−50.0	0.0	0.0	0.0	16.7	33.3	16.7	0.0	0.0	0.0	0.0	0.0	0.0
Pain	66.7	66.7	0.0	0.0	0.0	0.0	0.0	33.3	33.3	50.0	33.3	−16.7	0.0	0.0	0.0
Dyspnea	66.7	66.7	0.0	0.0	0.0	0.0	0.0	33.3	33.3	0.0	0.0	0.0	33.3	0.0	−33.3
Insomnia	33.3	66.7	33.3	33.3	0.0	−33.3	33.3	33.3	0.0	33.3	0.0	−33.3	0.0	0.0	0.0
Loss of appetite	66.7	66.7	0.0	0.0	0.0	0.0	0.0	0.0	0.0	0.0	0.0	0.0	33.3	33.3	0.0
Constipation	66.7	66.7	0.0	0.0	0.0	0.0	0.0	0.0	0.0	0.0	0.0	0.0	33.3	0.0	−33.3
Diarrhea	0.0	0.0	0.0	66.7	100.0	33.3	0.0	0.0	0.0	0.0	33.3	33.3	66.7	0.0	−66.7
Financial difficulties	66.7	66.7	0.0	0.0	0.0	0.0	0.0	33.3	33.3	0.0	33.3	33.3	33.3	0.0	−33.3

## Data Availability

The original contributions presented in this study are included in the article/Supplementary Materials. Further inquiries can be directed to the corresponding author.

## Supplementary Materials

The following supporting information can be downloaded at https://www.mdpi.com/article/10.3390/healthcare14213601/s1. Figure S1: Individual changes in waist-to-hip ratio from baseline to the end of the 12-week exercise intervention.; Figure S2: Changes from baseline to post-intervention of upper- and lower-limb flexibility. Table S1: Case-level description of the exercise intervention and achieved training dose.; Table S2: Individual physical outcome measures at baseline and after the 12-week exercise intervention. Table S3: CARE Checklist of information to include when writing a case report.

## Abbreviations

The following abbreviations are used in this manuscript:

**C****IFs**	Cachexia-inducing factors
**TME**	Tumor microenvironment
**QoL**	Quality of life
**BMI**	Body mass index
**ASCO**	American Society of Clinical Oncology
**CTCAE**	Common Terminology Criteria for Adverse Events
**ACSM**	American College of Sports Medicine
**ESSA**	Exercise & Sports Science Australia
**EORTC QLQ-C30**	European Organization for Research and Treatment of Cancer Quality of Life Questionnaire
**6MWT**	Six-minute walking test
