# Supplementary material for: Exercise Training in Patients with Established Cancer Cachexia: A Case Series
Source: Healthcare (Basel). 2026 Oct 23;14(21):3601. doi: 10.3390/healthcare14213601 (PMC13763131; doi:10.3390/healthcare14213601)
Supplement: Supplementary file 1 [file healthcare-14-03601-s001.zip › healthcare-4561044-supplementary.pdf]

**Figure S1**: Individual changes in waist-to-hip ratio from baseline to the end of the 12-week exercise intervention. Solid bars represent baseline values, whereas diagonally hatched bars represent values assessed after 12 weeks. Abbreviations: WHR, waist-to-hip ratio.

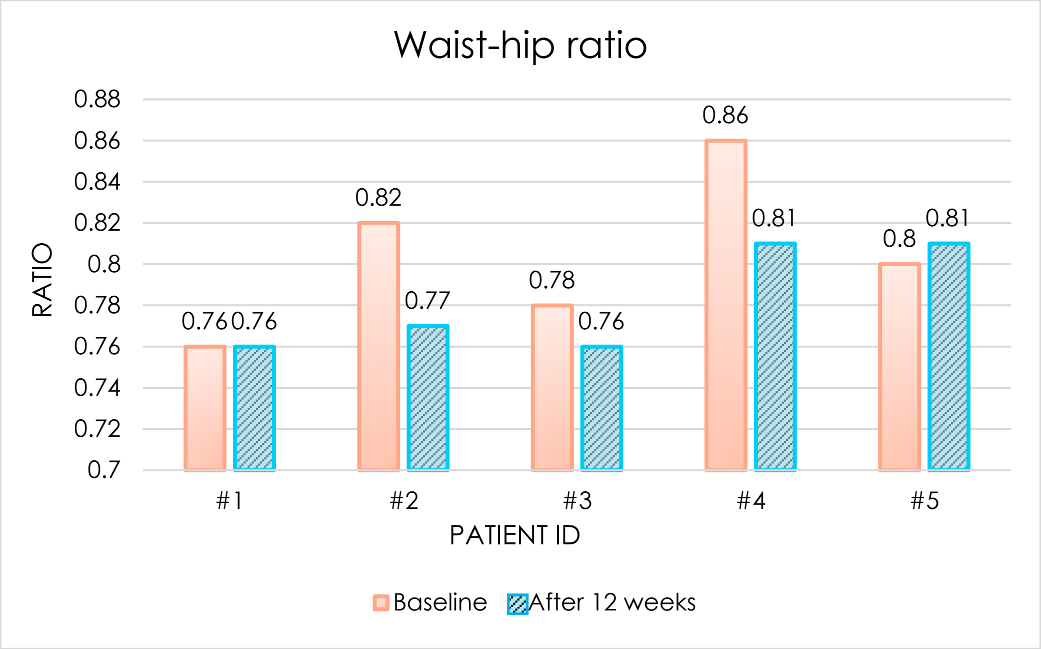

**Figure S2:** Individual changes in flexibility from baseline to the end of the 12-week exercise intervention. Panels (a) and (b) show right- and left-side lower-limb flexibility, respectively, assessed using the chair sit-and-reach test (cm), whereas panels (c) and (d) show right- and left-side upper-limb flexibility, respectively, assessed using the back-scratch test (cm). Solid bars represent baseline values, whereas diagonally hatched bars represent values assessed after 12 weeks. Abbreviations and units: cm, centimeters.

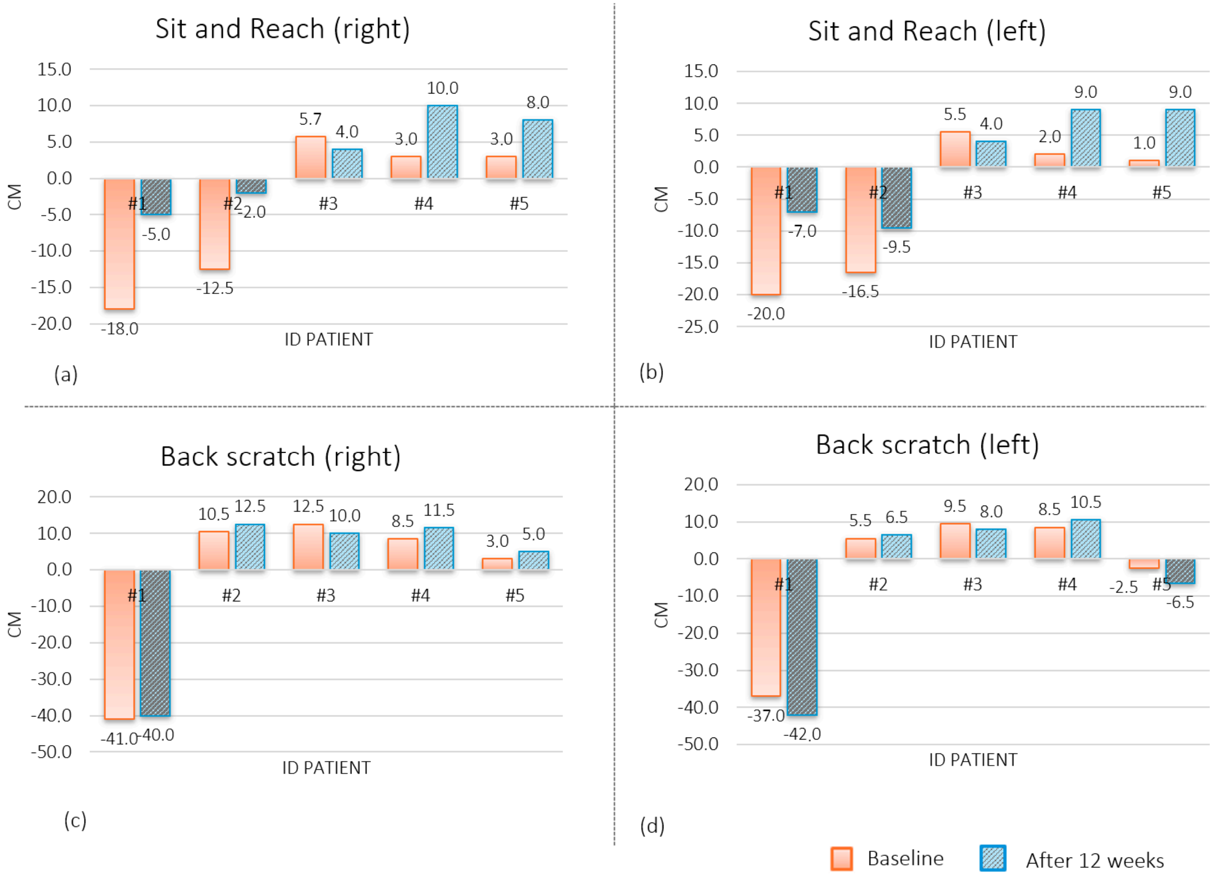

Table S1: Case-level description of the exercise intervention and achieved training dose.

| Intervention component | Patient #1 | Patient #2 | Patient #3 | Patient #4 | Patient #5 |
|---|---|---|---|---|---|
| Equipment for the exercise intervention | Aerobic training: cycle ergometer; resistance training: body weight, elastic bands, and isotonic machines | Aerobic training: cycle ergometer; resistance training: body weight, elastic bands, and isotonic machines | Aerobic training: cycle ergometer; resistance training: body weight, elastic bands, and isotonic machines | Aerobic training: cycle ergometer; resistance training: body weight, elastic bands, and isotonic machines | Aerobic training: cycle ergometer; resistance training: body weight, elastic bands, and isotonic machines |
| Provider and supervision | Exercise prescribed and individually supervised by trained exercise professionals | Exercise prescribed and individually supervised by trained exercise professionals | Exercise prescribed and individually supervised by trained exercise professionals | Exercise prescribed and individually supervised by trained exercise professionals | Exercise prescribed and individually supervised by trained exercise professionals |
| Setting | Facilities of the Sport Science at the University of Verona | Facilities of the Sport Science at the University of Verona | Facilities of the Sport Science at the University of Verona | Facilities of the Sport Science at the University of Verona | Facilities of the Sport Science at the University of Verona |
| Delivery | Individual, facility-based supervised sessions | Individual, facility-based supervised sessions | Individual, facility-based supervised sessions | Individual, facility-based supervised sessions | Individual, facility-based supervised sessions |
| Frequency and duration | 2 supervised sessions/week for 12 weeks | 2 supervised sessions/week for 12 weeks | 2 supervised sessions/week for 12 weeks | 2 supervised sessions/week for 12 weeks | 2 supervised sessions/week for 12 weeks |
| Attendance/adherence recording | Prospectively recorded by exercise professionals using an individual paper-based exercise log; attendance and completion of the prescribed dose documented at each session | Prospectively recorded by exercise professionals using an individual paper-based exercise log; attendance and completion of the prescribed dose documented at each session | Prospectively recorded by exercise professionals using an individual paper-based exercise log; attendance and completion of the prescribed dose documented at each session | Prospectively recorded by exercise professionals using an individual paper-based exercise log; attendance and completion of the prescribed dose documented at each session | Prospectively recorded by exercise professionals using an individual paper-based exercise log; attendance and completion of the prescribed dose documented at each session |
| Behavioral component | No structured behavioral intervention; verbal encouragement to remain physically active on non-supervised days | No structured behavioral intervention; verbal encouragement to remain physically active on non-supervised days | No structured behavioral intervention; verbal encouragement to remain physically active on non-supervised days | No structured behavioral intervention; verbal encouragement to remain physically active on non-supervised days | No structured behavioral intervention; verbal encouragement to remain physically active on non-supervised days |
| Concomitant care | Usual oncological treatment and nutritional care continued throughout the intervention | Usual oncological treatment and nutritional care continued throughout the intervention | Usual oncological treatment and nutritional care continued throughout the intervention | Usual oncological treatment and nutritional care continued throughout the intervention | Usual oncological treatment and nutritional care continued throughout the intervention |
| Detailed exercise intervention | Warm-up<br>• 5 min of 5-6 dynamic mobility/stretching exercises between shoulder circles, arm swings, trunk rotations, hip mobility, knee flexion–extension, ankle mobilization | Warm-up<br>• 5 min of 5–6 dynamic mobility/stretching exercises including shoulder circles, arm swings, trunk rotations, hip mobility, knee flexion– | Warm-up<br>• 5 min of 5–6 dynamic mobility/stretching exercises including shoulder circles, arm swings, trunk rotations, hip mobility, knee flexion– | Warm-up<br>• 5 min of 5–6 dynamic mobility/stretching exercises including shoulder circles, arm swings, trunk rotations, hip mobility, knee flexion– | Warm-up<br>• 5 min of 5–6 dynamic mobility/stretching exercises including shoulder circles, arm swings, trunk rotations, hip mobility, knee flexion– |

| Intervention component | Patient #1 | Patient #2 | Patient #3 | Patient #4 | Patient #5 |
|---|---|---|---|---|---|
| | Aerobic component<br>• Cycle ergometer<br>• Initial duration: 10 min/session<br>• Prescribed intensity: RPE 3–5, modified Borg CR-10 scale<br>• Achieved intensity: RPE 4-5<br>• Planned progression: duration increased by 5 min/week, according to a predefined progression schedule, from 10 to 15 and then 20 min/session<br>• Progression was implemented provided that perceived exertion remained within the prescribed RPE range<br>• Maximum duration achieved: 20 min/session<br><br>Resistance training<br>• 5 exercises/session targeting major muscle groups<br>• Exercise pool: box squat, row, abdominal exercise, calf raise, biceps curl, chest press, lat pulldown, leg press, leg curl, and leg extension<br>• No fixed exercise combination was assigned. Five exercises per session were selected from the predefined exercise pool according to functional capacity, symptoms, clinical condition, and available range of motion. Specific exercises performed at each attended session were recorded in the individual exercise logs<br>• Initial volume: 2 × 8 repetitions | extension, and ankle mobilization<br><br>Aerobic component<br>• Cycle ergometer<br>• Initial duration: 10 min/session<br>• Prescribed intensity: RPE 3–5, modified Borg CR-10 scale<br>• Achieved intensity: RPE 3–5<br>• Planned progression: duration increased by 5 min/week, according to a predefined progression schedule, from 10 to 15 and then 20 min/session<br>• Progression was implemented provided that perceived exertion remained within the prescribed RPE range<br>• Maximum duration achieved: 20 min/session<br><br>Resistance training<br>• 5 exercises/session targeting major muscle groups<br>• Exercise pool: box squat, row, abdominal exercise, calf raise, biceps curl, chest press, lat pulldown, leg press, leg curl, and leg extension<br>• No fixed exercise combination was assigned. Five exercises per session were selected from the predefined exercise pool according to functional | extension, and ankle mobilization<br><br>Aerobic component<br>• Cycle ergometer<br>• Initial duration: 15 min/session<br>• Prescribed intensity: RPE 3–5, modified Borg CR-10 scale<br>• Achieved intensity: RPE 3–5<br>• Planned progression: duration increased according to a predefined progression schedule, from 15 to 20 min/session<br>• Progression was implemented provided that perceived exertion remained within the prescribed RPE range<br>• Maximum duration achieved: 20 min/session<br><br>Resistance training<br>• 5 exercises/session targeting major muscle groups<br>• Exercise pool: box squat, row, abdominal exercise, calf raise, biceps curl, chest press, lat pulldown, leg press, leg curl, and leg extension<br>• No fixed exercise combination was assigned. Five exercises per session were selected from the predefined exercise pool according to functional capacity, symptoms, clinical | extension, and ankle mobilization<br><br>Aerobic component<br>• Cycle ergometer<br>• Initial duration: 10 min/session<br>• Prescribed intensity: RPE 3–5, modified Borg CR-10 scale<br>• Achieved intensity: RPE 3–5<br>• Planned progression: duration increased by 5 min/week, according to a predefined progression schedule, from 10 to 15 and then 20 min/session<br>• Progression was implemented provided that perceived exertion remained within the prescribed RPE range<br>• Maximum duration achieved: 20 min/session<br><br>Resistance training<br>• 5 exercises/session targeting major muscle groups<br>• Exercise pool: box squat, row, abdominal exercise, calf raise, biceps curl, chest press, lat pulldown, leg press, leg curl, and leg extension<br>• No fixed exercise combination was assigned. Five exercises per session were selected from the predefined exercise pool according to functional | extension, and ankle mobilization<br><br>Aerobic component<br>• Cycle ergometer<br>• Initial duration: 15 min/session<br>• Prescribed intensity: RPE 3–5, modified Borg CR-10 scale<br>• Achieved intensity: RPE 3–5<br>• Planned progression: duration increased according to a predefined progression schedule, from 15 to 20 min/session<br>• Progression was implemented provided that perceived exertion remained within the prescribed RPE range<br>• Maximum duration achieved: 20 min/session<br>Resistance training<br>• 5 exercises/session targeting major muscle groups<br>• Exercise pool: box squat, row, abdominal exercise, calf raise, biceps curl, chest press, lat pulldown, leg press, leg curl, and leg extension<br>• No fixed exercise combination was assigned. Five exercises per session were selected from the predefined exercise pool according to functional capacity, symptoms, clinical condition, and available |

| Intervention component | Patient #1 | Patient #2 | Patient #3 | Patient #4 | Patient #5 |
|---|---|---|---|---|---|
| | • Planned progression: 2×8 → 2×10 → 2×12 → 3×8 → 3×10 → 3×12<br>• Resistance-training volume was progressed according to a predefined schedule every 2 weeks<br>• Progression was performed only when perceived exertion remained within the prescribed RPE range<br>• Maximum achieved: 5 exercises, 3 × 12 repetitions<br>• Achieved intensity: RPE 3–5<br>• Rest between sets: 1–2 min<br>• External load was individually adjusted to maintain the prescribed RPE range; absolute loads were not systematically recorded.<br>• Exercise execution and range of motion were individualized according to functional capacity, clinical condition, symptoms, and coordination<br>Cool-down<br>• 5 min; 5-6 static stretching exercises; 30 seconds: calf, hamstring, quadriceps, chest, and upper-back stretching | capacity, symptoms, clinical condition, and available range of motion. Specific exercises performed at each attended session were recorded in the individual exercise logs<br>• Initial volume: 2 × 10 repetitions<br>• Planned progression: 2×10 → 2×12 → 3×8 → 3×10 → 3×12<br>• Resistance-training volume was progressed according to a predefined schedule every 2 weeks<br>• Progression was performed only when perceived exertion remained within the prescribed RPE range<br>• Maximum achieved: 5 exercises, 3 × 12 repetitions<br>• Achieved intensity: RPE 3–5<br>• Rest between sets: 1–2 min<br>• External load was individually adjusted to maintain the prescribed RPE range; absolute loads were not systematically recorded.<br>• Exercise execution and range of motion were individualized according to functional capacity, clinical condition, symptoms, and coordination<br>Cool-down<br>• 5 min of 5–6 static stretching exercises, held for approximately 30 seconds, including calf, | condition, and available range of motion. Specific exercises performed at each attended session were recorded in the individual exercise logs<br>• Initial volume: 2 × 10 repetitions<br>• Planned progression: 2×10 → 2×12 → 3×8 → 3×10 → 3×12<br>• Resistance-training volume was progressed according to a predefined schedule every 2 weeks<br>• Progression was performed only when perceived exertion remained within the prescribed RPE range<br>• Maximum achieved: 5 exercises, 3 × 12 repetitions<br>• Achieved intensity: RPE 3–5<br>• Rest between sets: 1–2 min<br>• External load was individually adjusted to maintain the prescribed RPE range; absolute loads were not systematically recorded.<br>• Exercise execution and range of motion were individualized according to functional capacity, clinical condition, symptoms, and coordination<br>Cool-down<br>• 5 min of 5–6 static stretching exercises, held for approximately 30 seconds, including calf, hamstring, quadriceps, | capacity, symptoms, clinical condition, and available range of motion. Specific exercises performed at each attended session were recorded in the individual exercise logs<br>• Initial volume: 2 × 10 repetitions<br>• Planned progression: 2×10 → 2×12 → 3×8 → 3×10 → 3×12<br>• Resistance-training volume was progressed according to a predefined schedule every 2 weeks<br>• Progression was performed only when perceived exertion remained within the prescribed RPE range<br>• Maximum achieved: 5 exercises, 3 × 12 repetitions<br>• Achieved intensity: RPE 3–5<br>• Rest between sets: 1–2 min<br>• External load was individually adjusted to maintain the prescribed RPE range; absolute loads were not systematically recorded.<br>• Exercise execution and range of motion were individualized according to functional capacity, clinical condition, symptoms, and coordination<br>Cool-down<br>• 5 min of 5–6 static stretching exercises, held for approximately 30 seconds, including calf, | range of motion. Specific exercises performed at each attended session were recorded in the individual exercise logs<br>• Initial volume: 2 × 10 repetitions<br>• Planned progression: 2×10 → 2×12 → 3×8 → 3×10 → 3×12<br>• Resistance-training volume was progressed according to a predefined schedule every 2 weeks<br>• Progression was performed only when perceived exertion remained within the prescribed RPE range<br>• Maximum achieved: 5 exercises, 3 × 12 repetitions<br>• Achieved intensity: RPE 3–5<br>• Rest between sets: 1–2 min<br>• External load was individually adjusted to maintain the prescribed RPE range; absolute loads were not systematically recorded.<br>• Exercise execution and range of motion were individualized according to functional capacity, clinical condition, symptoms, and coordination<br>Cool-down<br>• 5 min of 5–6 static stretching exercises, held for approximately 30 seconds, including calf, hamstring, quadriceps, |

| Intervention component | Patient #1 | Patient #2 | Patient #3 | Patient #4 | Patient #5 |
|---|---|---|---|---|---|
| | | hamstring, quadriceps, chest, shoulder, and upper-back stretching | chest, shoulder, and upper-back stretching | hamstring, quadriceps, chest, shoulder, and upper-back stretching | chest, shoulder, and upper-back stretching |
| Individual adaptations | Additional monitoring of RPE, fatigue, and cardiovascular symptoms (e.g., palpitations) due to cardiomyopathy; upper-body exercises were adapted to the available shoulder range of motion following breast cancer surgery. | No specific adaptations required | Following abdominal surgery, exercises involving the abdominal region were preferentially performed using isometric contractions to limit mechanical stress | No specific adaptations required | No specific adaptations required |
| Planned progression vs dose modification | Scheduled progression in aerobic duration and resistance volume was part of the planned prescription and was distinguished from unplanned dose modifications due to symptoms or exercise intolerance | Scheduled progression in aerobic duration and resistance volume was part of the planned prescription and was distinguished from unplanned dose modifications due to symptoms or exercise intolerance | Scheduled progression in aerobic duration and resistance volume was part of the planned prescription and was distinguished from unplanned dose modifications due to symptoms or exercise intolerance | Scheduled progression in aerobic duration and resistance volume was part of the planned prescription and was distinguished from unplanned dose modifications due to symptoms or exercise intolerance | Scheduled progression in aerobic duration and resistance volume was part of the planned prescription and was distinguished from unplanned dose modifications due to symptoms or exercise intolerance |
| Unplanned dose reductions/early termination | None | None | None | None | None |
| Attendance | 16/24 sessions (66.7%) | 13/24 sessions (54.2%) | 23/24 sessions (95.8%) | 21/24 sessions (87.5%) | 20/24 sessions (83.3%) |
| Adherence during attended sessions | 100%; prescribed exercise dose completed, with no unplanned dose reduction or early termination | 100%; prescribed exercise dose completed, with no unplanned dose reduction or early termination | 100%; prescribed exercise dose completed, with no unplanned dose reduction or early termination | 100%; prescribed exercise dose completed, with no unplanned dose reduction or early termination | 100%; prescribed exercise dose completed, with no unplanned dose reduction or early termination |

Table S2. Individual physical outcome measures at baseline and after the 12-week exercise intervention.

| Variables | *Case #1* | | | *Case #2* | | | *Case #3* | | | *Case #4* | | | *Case #5* | | |
|---|---|---|---|---|---|---|---|---|---|---|---|---|---|---|---|
| | *T0* | *T1* | *Δ score (T1–T0)* | *T0* | *T1* | *Δ score (T1–T0)* | *T0* | *T1* | *Δ score (T1–T0)* | *T0* | *T1* | *Δ score (T1–T0)* | *T0* | *T1* | *Δ score (T1–T0)* |
| Anthropometric measures | | | | | | | | | | | | | | | |
| Body weight (kg) | 55.1 | 55.7 | *+0.6* | 45.6 | 45.8 | *+0.2* | 52.7 | 46.7 | *-6* | 42.6 | 45.1 | *+2.5* | 52.9 | 52.8 | *-0.1* |
| Body mass index (kg/m$^2$) | 19.7 | 19.9 | *+0.2* | 16.8 | 16.9 | *+0.1* | 20.9 | 18.5 | *-2.4* | 18.7 | 19.8 | *+1.1* | 19.2 | 19.1 | *-0.1* |
| Waist (cm) | *67* | *66.5* | *-0.5* | *66* | *63.5* | *-2.5* | *71* | *64* | *-7* | *68.5* | *67* | *-1.5* | *73* | *74.5* | *+1.5* |
| Hip (cm) | *88* | *87* | *-1* | *80.5* | *82.5* | *+2* | *91.5* | *84.5* | *-7* | *79.5* | *83* | *+3.5* | *91* | *92* | *+1* |
| Waist-hip ratio | 0.76 | 0.76 | *0* | 0.82 | 0.77 | *-0.05* | 0.78 | 0.76 | *-0.02* | 0.86 | 0.81 | *-0.05* | 0.80 | 0.81 | *+0.01* |
| Body composition (Kg) | | | | | | | | | | | | | | | |
| Fat mass | *8.3* | *10.2* | *+1.9* | *7.7* | *5.1* | *-2.6* | *16.6* | *11.6* | *-5* | *5.6* | *6.8* | *+1.2* | *13.9* | *10.8* | *-3.1* |
| Fat free mass | *46.8* | *45.5* | *-1.3* | *37.9* | *40.7* | *+2.8* | *36.1* | *35.1* | *-1* | *37.0* | *38.3* | *+1.3* | *39.1* | *41.9* | *+2.8* |
| Chair sit and reach (cm) | | | | | | | | | | | | | | | |
| Right leg | -18 | -5 | +13 | -12.5 | -2 | +10.5 | 5.5 | 4 | -1.5 | 3 | 10 | +7 | 3 | 8 | +5 |
| Left leg | -20 | -7 | +13 | -16.5 | -9.5 | +7 | 5.5 | 4 | -1.5 | 2 | 9 | +7 | 1 | 9 | +8 |
| Back scratch (cm) | | | | | | | | | | | | | | | |
| Right arm | -41 | -40 | +1 | 10.5 | 12.5 | +2 | 12.5 | 10 | -2.5 | 8.5 | 11.5 | +3 | 3 | 5 | +2 |
| Left arm | -37 | -42 | -5 | 5.5 | 6.5 | +1 | 9.5 | 8 | -1.5 | 8.5 | 10.5 | +2 | -2.5 | -6.5 | -4 |
| Handgrip (kg) | | | | | | | | | | | | | | | |
| Right arm | 7.5 | 23 | +15.5 | 25 | 29 | +4 | 25 | 25.5 | +0.5 | 26 | 26.5 | +0.5 | 27 | 28 | +1 |
| Left arm | 16.5 | 20 | +3.5 | 20.5 | 24 | +3.5 | 21 | 21 | 0 | 24.5 | 26.5 | +2 | 27 | 28 | +1 |
| Six-minute walking test (m) | 409.4 | 450.3 | +40.9 | 506.8 | 545 | +38.2 | 566.9 | 600 | +33.1 | 514 | 587.2 | +73.2 | 546.9 | 600 | +53.1 |
